# Gut Feelings: The Psychological Impact of Inflammatory Bowel Disease

**DOI:** 10.3390/jcm12123867

**Published:** 2023-06-06

**Authors:** Antonio M Caballero-Mateos

**Affiliations:** 1 Gastroenterology and Hepatology Department, San Cecilio University Hospital, 18012 Granada, Spain; ogy1492@hotmail.com; 2 Internal Medicine Department, Hospital Comarcal “Santa Ana”, 18600 Motril, Spain

**Keywords:** inflammatory bowel disease, Crohn’s disease, ulcerative colitis, anxiety, depression

Inflammatory bowel disease (IBD) is a chronic and debilitating condition that impacts a substantial number of individuals globally. It manifests as inflammation within the gastrointestinal tract, resulting in a diverse array of symptoms such as abdominal pain, diarrhea, and rectal bleeding. The primary subtypes of IBD are Crohn’s disease (CD) and ulcerative colitis (UC).

The complex and multifactorial relationship between IBD and mental health has become increasingly evident. Individuals with IBD are at increased risk of developing depression and anxiety, which can further worsen their symptoms and overall well-being. Psychological stress has been identified as a prevalent factor in IBD with detrimental effects on patients’ quality of life and potentially impacting disease progression. Moreover, fatigue is a significant clinical concern in IBD, affecting a substantial number of patients even during clinical remission. Fatigue not only impairs quality of life but also hampers work productivity and overall functioning. The management of IBD has faced considerable challenges during the COVID-19 pandemic, with restriction of hospitalizations and unprecedented redeployment of health care resources. This situation has been associated with increased psychological distress, particularly among patients with chronic diseases, including IBD.

In this editorial, we aim to emphasize the importance of addressing psychological health in patients with IBD. We will explore the prevalence of depression, anxiety, and fatigue in these patients, shedding light on their impact on quality of life. Additionally, we will discuss the use of patient-reported outcomes (PROs) in IBD research and clinical evaluation, which have significantly contributed to our understanding of the psychological burden associated with the disease. Furthermore, we will examine the impact of the COVID-19 pandemic on the mental health and behavior of IBD patients. Finally, we will explore the future of psychological health in clinical practice and strategies to better address this crucial aspect of patient care.

Anxiety and depression are common psychological symptoms among patients with IBD. Anxiety symptoms have a prevalence of 38.5%, and depression symptoms 22.8% [1], as seen in other gastrointestinal conditions such as Functional Dyspepsia [2]. Sex differences have been observed in psychological symptoms, sleep quality, and overall quality of life. For instance, anxiety symptoms were more prevalent in female IBD patients (38.5% vs. 22.8% in males). Similarly, sleep disturbances were more common in women (51.3% vs. 37.9% in men), while male IBD patients demonstrated higher scores for quality of life. Intestinal permeability has been suggested as a potential factor contributing to depression in IBD [3]. Recent research has highlighted the role of gut permeability markers, such as zonulin and lipopolysaccharide binding protein (LBP), which were found to be significantly elevated in patients with depression. These findings suggest potential common pathophysiological mechanisms involving microbiota, intestinal permeability, and inflammation in both conditions.

Patient-reported outcomes (PROs) have become increasingly important in clinical trials and research studies. They provide valuable information about patients’ experiences with their disease and treatment, which can help inform clinical decision-making. The inclusion of PROs in IBD research has enhanced our understanding of the psychological impact of the disease and underscores the significance of addressing this aspect in patient care. Fatigue, a prevalent and debilitating symptom in IBD, is often overlooked and challenging to treat. Up to 80% of patients with active disease and 50% of those in remission report experiencing fatigue [4]. Furthermore, 60% of IBD patients report fatigue as a symptom that is closely associated with gastrointestinal symptoms such as nausea and vomiting, abdominal pain, and diarrhea. The pathophysiology of fatigue in IBD is multifactorial, involving inflammatory processes, psychosocial comorbidities, nutritional deficiencies, metabolic alterations, lifestyle factors, and changes in the gut microbiota. A recent multicenter study conducted at five academic hospitals in Korea involving 147 IBD patients aimed to assess the prevalence of fatigue using the validated Korean version of the Multidimensional Fatigue Inventory (MFI-K) [5]. Among 97 UC patients and 50 CD patients, the mean total MFI-K score was 59.0 ± 5.5, which corresponded to a moderate-to-severe level of fatigue. The study also found associations between depression and reduced motivation subscale scores, as well as between a history of IBD-related surgery and higher total MFI-K scores. Notably, the use of biologic medications was associated with lower total MFI-K scores. Disability is a PRO that is defined by an objective limitation of function, and it is becoming a more important goal of IBD treatment. Disability is another crucial patient-reported outcome that reflects objective limitations in functioning. The Inflammatory Bowel Disease-Disk Tool, a visual instrument consisting of ten items, has been developed to assess disability in IBD patients [6]. This tool explores various aspects of patients’ lives, including abdominal pain, body image, education and work, emotions, energy, interpersonal interactions, joint pain, defecation regulation, sexual functions, and sleep. The IBD-Disk Tool has demonstrated high internal consistency and test–retest reliability, with higher scores correlating with increased disease activity. Female patients consistently exhibited higher disability scores, regardless of other disease characteristics, while the presence of extraintestinal manifestations was associated with increased disability in both UC and CD patients.

The COVID-19 pandemic has had a considerable impact on IBD patients by limiting their access to medical services due to restrictions and the reorganization of the healthcare systems, which affects their quality of life. Despite initial concerns about their vulnerability to SARS-CoV-2 infection, it has been found that the risk of severe COVID-19 is not increased in IBD patients [7]. Nevertheless, the prevalence of depression and anxiety symptoms increased in IBD patients during the pandemic [8]. High anxiety and depression levels were associated with a slight decrease in medication adherence. Simulation models analyzing the effects of the pandemic on IBD care have highlighted disruptions in patient behaviors and medical services, including reduced clinic visits, delays in diagnosis, and decreased use of healthcare services [9]. These models emphasize the importance of timely interventions to mitigate the initial impact and address disruptions effectively.

It is crucial to acknowledge the potential mental health implications for patients with IBD and provide appropriate support and resources. Healthcare providers should consider the holistic needs of patients, including their mental well-being, and ensure access to mental health services, telemedicine options, patient education initiatives, and other resources to help manage the psychosocial impact of the pandemic. Research on acceptance and commitment therapy (ACT) interventions for stress in IBD populations has shown promise [10]. The study found that brief and telehealth ACT interventions with blended delivery components including workbooks and phone consultations are feasible for people with long-term health conditions and, specifically, IBD. Additionally, non-standard pharmacological treatments have demonstrated potential benefits in managing psychological symptoms in IBD patients. A double-blind, randomized prospective trial found that giving low-dose S-ketamine during surgery could help alleviate mild-to-moderate depressive symptoms and postoperative pain in patients with CD undergoing bowel resection without worsening their safety [11]. Another study found that a single dose of ferric carboxymaltose improved the quality of life of patients with IBD and iron deficiency without anemia at 1 month after treatment [12].

It is important to integrate psychological treatments into a comprehensive care plan that includes medical management, dietary interventions, and other aspects of IBD treatment. Collaboration between healthcare providers, including gastroenterologists, psychologists, and other mental health professionals, is essential. As our understanding of the disease deepens and our treatment options expand, it is our moral imperative to prioritize the well-being of our patients and strive for an improved quality of life. This includes addressing the often overlooked aspects of mental health, which play a crucial role in their overall well-being.
